# Efficacy of bisphosphonates in the treatment of femoral head osteonecrosis: A PRISMA-compliant meta-analysis of animal studies and clinical trials

**DOI:** 10.1038/s41598-018-19884-z

**Published:** 2018-01-23

**Authors:** Donghai Li, Zhouyuan Yang, Zhun Wei, Pengde Kang

**Affiliations:** Department of Orthopaedics, West China Hospital, Sichuan University, 37# Wainan Guoxue Road, Chengdu, 610041 People’s Republic of China

## Abstract

This study aimed to determine whether bisphosphonates exert an effect on preventing femoral head collapse after osteonecrosis of the femoral head (ONFH) in an animal model and in clinical trials. A systematic literature search was performed for studies published up to January 2017. Twenty-three articles (16 animal studies, seven clinical trials) were included in the meta-analysis. We found that the bisphosphonate group obtained significant improvement in epiphyseal quotients (MD = 15.32; 95% CI, 9.25–21.39) and provided better performance on bone volume (SMD = 1.57; 95% CI, 0.94–2.20), trabecular number (SMD = 1.30; 95% CI, 0.80–1.79), trabecular thickness (SMD = 0.77; 95% CI, 0.10–1.43) and trabecular separation (SMD = −1.44; 95% CI, −1.70 to −0.58) in the animal model. However, the bisphosphonate group did not achieve better results in pain score, Harris score, the occurrence rate of femoral head collapse, or total hip arthroplasty in the clinical trials. In conclusion, despite bisphosphonates significantly improving bone remodeling outcomes in animal models, no significant efficacy was observed in the treatment of ONFH in the clinical studies. Further studies are required to solve the discordant outcomes between the animal and clinical studies.

## Introduction

Osteonecrosis of the femoral head (ONFH) is a common debilitating disease that occurs in young and middle-aged adults^1,2^. In fact, children also suffer from ONFH with an incidence of 8.5–21 per 100 000, but in this population, it is called Perthes disease^3,4^. Although the progressions of adult ONFH and Perthes disease differ, both conditions result in femoral head deformity or collapse. Therefore, preventing femoral head collapse is a significant treatment goal^5,6^. The pathogenesis of ONFH remains unclear, but an imbalance of bone metabolism is considered one of the most important causes^7^. When ONFH occurs, bone formation fails to keep pace with bone resorption, resulting in low bone mineral density in the femoral head and the progression to collapse^8^. Therefore, clinicians must take measures to reduce bone resorption and improve osteogenesis when treating ONFH.

Bisphosphonates are a class of drugs that can bind to the bone and inhibit osteoclast activity by reducing bone resorption^9–11^. They are usually used to treat diseases involving bone resorption progression, such as osteoporosis, Paget’s disease, and fibrous dysplasia^11–13^. Bisphosphonates has also been considered a promising medication for early ONFH and preventing femoral head collapse^14–17^. However, the efficiency of this kind of drug in both animal studies^18,19^ and clinical trials^20,21^ remains controversial. Furthermore, a meta-analysis of a small number of clinical studies reported that the use of bisphosphonates cannot prevent femoral head collapse or delay total hip replacement after ONFH^22^. Consequently, the use of bisphosphonates in the early stage of ONFH seems to involve some challenges.

To evaluate the effect of bisphosphonates on preventing femoral head collapse after osteonecrosis, we identified all related animal studies and clinical trials from the electronic database and conducted this meta-analysis comprehensively to judge whether bisphosphonates should be recommended to ONFH patients and are worthy of further study.

## Materials and Methods

This meta-analysis conformed to the Preferred Reporting Items for Systematic Reviews and Meta-Analyses statement^23^.

### Search strategy

The study’s search protocol was developed on December 20, 2016. An electronic search was conducted online to identify relevant studies published up to January 2017 in PubMed; Ovid MEDLINE(R) (1946 to present with daily update); all EBM reviews; the ISI Web of Science; Academic Search Premier, and MEDLINE in EBSCO; Cochrane Library databases; CBM; and CNKI databases using the following terms: (ibandronate or alendronate or bisphosphonate or zoledronate or pamidronate or clodronate) AND (osteonecrosis of the femoral head or femoral head necrosis or Perthes disease) in all fields. In addition, the reference lists of the retrieved articles were manually searched for further pertinent studies. Furthermore, we contacted the study authors for the raw data and to complete the search strategy whenever possible. The two investigators independently selected potential eligible studies and any discrepancy between them was resolved by consensus.

### Data extraction

The data collection was conducted by two investigators independently and the result was checked by a third investigator. Discrepancies were settled by group discussion. Collected data included the first author’s surname, publication year, study location, study design, sample size, type of medicine or dose range, and route of medication delivery. For the animal studies, we extracted the characteristics of the animal models including species, animal age, weight, and sex; and outcomes including mean epiphyseal quotients (EQ), trabecular bone volume (BV), trabecular separation (TS), trabecular thickness (TT), and trabecular number (TN). When various methods were presented and more than two groups were analyzed in a single study, the data were assessed as two comparisons of those exposed to bisphosphonates if necessary. For clinical trials, we recorded pain scores, Harris scores, and femoral head collapse and THA occurrence rates.

### Assessment of methodological quality

Two reviewers independently assessed the methodology of the included animal studies using updated Stroke Therapy Academic Industry Roundtable recommendations^24^ and that of the clinic studies using the modified Jadad scale^25^. The methodological quality of each individual study was scored against the following criteria: sample size calculation; inclusion and exclusion criteria; randomization; allocation concealment; reporting of objects excluded from the analysis; blinded assessment of the outcomes; and report of potential conflicts of interest and study funding. Each item was allocated one point for a quantitative appraisal of overall quality of the individual studies. Each animal study was given a quality score out of a possible total of seven points and the group median was calculated. The modified Jadad scale evaluated the clinical studies in terms of randomization (2 points); concealment of allocation (2 points); double blinding (2 points); and total withdrawals and dropouts (1 point). Clinical studies achieving a score of >4 points were considered of high quality.

### Statistical analysis

This meta-analysis was conducted using Review Manager Software (Revman 5.3, Cochrane Collaboration, Oxford, United Kingdom). For the animal studies, we divided them into Perthes model and mature ONFH model subgroups and analyzed the pooled results. The relative risk (RR) was used to measure the dichotomous outcomes, while the mean difference (MD) was used to analyze continuous outcomes, both with 95% confidence intervals (CI). When the parameter was measured using different methods, standard mean difference (SMD) was used to compare continuous outcomes (e.g. BV, TT, TN, and TS). Chi squared tests and the I^2^ statistic were used to evaluate statistical heterogeneity. An I^2^ > 50% was considered to indicate significantly statistical heterogeneity and the random-effect or fixed-effect model was used. Publication bias was visually examined using funnel plots. Values of P < 0.05 were considered statistically significant. The sensitivity analysis was performed to explore the impact of an individual study by the exclusion of one study each time. Publication bias was visually examined using funnel plots.

## Results

### Search results and study characteristics

**Figure 1:**
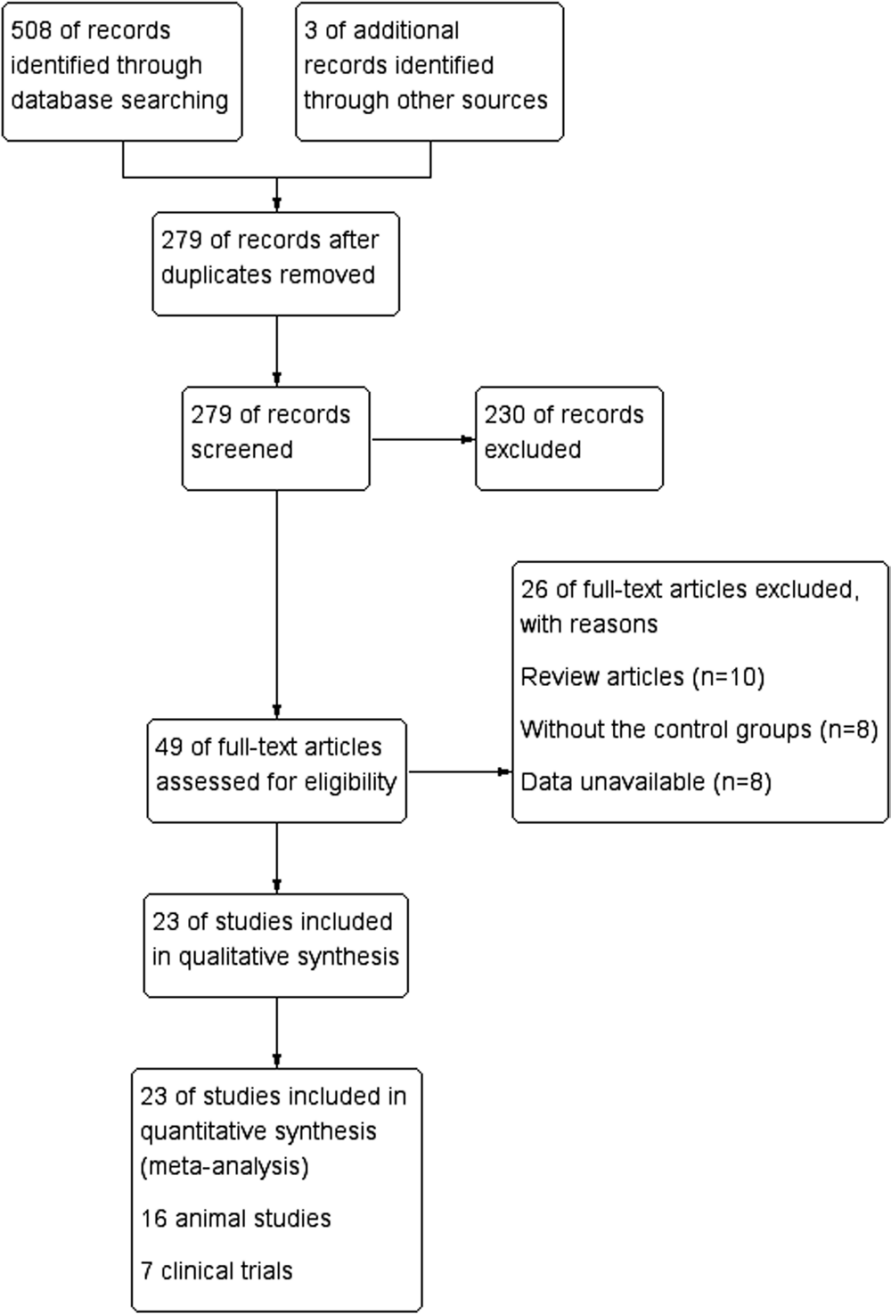
Flow chart of study selection.

As shown in Fig. 1, a total of 508 potentially relevant articles were identified from the databases. Of them, 279 were screened. After a title and abstract screen, 230 were excluded. A total of 49 full-text articles were assessed for eligibility, but 26 were excluded for different reasons (10 review articles, eight studies lacked a control group, and data for eight studies were unavailable). The remaining 23 articles including 16 animal studies^16–19,26–37^ and seven human studies^14,15,20,21,38–40^ were passed for synthetic evaluation for this meta-analysis. A summary of selected studies is shown in Table 1, while the basic techniques of the included studies are shown in Table 2. As Tables 3 and 4 present, the median quality score of the reported animal studies was 4 (range, 3–6), while the median of modified Jadad score of the clinical studies was 5 (range, 4–7).

**Table 1:** Summary of the basic information.

**Author**	**Year**	**Country**	**Study Object**	**SG (n/hip)**	**Age**	**Sex,M/F**	**CG (n/hip)**	**Age**	**Sex,M/F**
Animal studies									
Kim HK(1)	2005	USA	piglet	8	4–5 w	male	8	4–5 w	male
Aya-ay J	2007	Germany	piglet	5	6–9 w	male	4	6–9 w	male
Vandermeer JS	2011	USA	piglet	5	6–8 w	male	5	6–8 w	male
Kim HK (2)	2014	USA	piglet	6	6–8 w	male	6	6–8 w	male
Cheng TL	2014	Australia	piglet	4	6–8 w	male	4	6–8 w	male
Zou Y	2015	USA	piglet	6	2–3 w	male	9	2–3 w	male
Aruwajoye OO	2016	USA	Piglet	10	5–8 w	male	10	5–8 w	Male
Little DG(1)	2003	Australia	Rat	16	14 w	female	8	14 w	Female
Little DG(2)	2005	Australia	Rat	32	4 w	female	16	4 w	Female
Fan M	2012	China	Rat	8	NR	male	8	NR	Male
Xu XL	2015	China	Rat	10	8 w	male	10	8 w	male
Hofstaetter JG	2009	USA	Rabbit	15	10–12 m	male	15	10–12 m	Male
Xu XL	2015	China	Rat	10	8 w	male	10	8 w	male
Xie XW	2013	China	Rat	30	8 w	unlimited	30	8 w	unlimited
Tan G	2013	China	Rat	60	8 w	unlimited	60	8 w	unlimited
Xin DS(1)	2014	China	Rat	15	NR	male	15	NR	male
Xin DS(2)	2014	China	Rat	15	NR	male	15	NR	male
Clinical studies									
Wang CJ	2008	Taiwan	Human	23/30	35.7 ± 4.7y	13/10	25/30	38.6 ± 12.6y	20/5
Chen CH	2012	Taiwan	Human	26/32	48.4 ± 11.4y	22/4	26/33	44.2 ± 9.2y	19/7
Nishii T	2006	Japan	Human	14/20	48y	07/7	Aug-13	36y	07/1
Lai KA	2005	Taiwan	Human	20/29	42.6y	NR	20/25	42.4y	NR
Lee YK	2015	Korea	Human	55/55	43.8 ± 11.8y	39/1	55/55	45.2 ± 11.6y	41/1
Kang P	2012	China	Human	39/55	43.8y	6	40/52	45.3y	4
Gao yan	2016	China	Human	32/32	23–55y	37/18	32/32	23–55y	35/17
						21/11			22/10

SG: study group or bisphosphonates group; CG: control group. unlimited means animal gender is not limited. n/hip: animal number/hip number. w- weeks. y: years old. m: month. NR: not report.

**Table 2:** Detail information of the research technique.

**Author**	**Kind of ONFH**	**Study design**	**Drug delivery**	**Dosage**	**Study period**
Animal studies					
Kim HK(1)	Traumatic	Ibandronate VS placebo	Subcutaneous	44.4 μg/kg,3 × per/w × 6 w	8 weeks
Aya-ay J	Traumatic	Ibandronate VS placebo	intraosseous	0.28 mg or 0.56 mg, once	7 weeks
Vandermeer JS	Traumatic	Ibandronate VS placebo	intraosseous	0.56 mg, once	8 weeks
Kim HK (2)	Traumatic	Ibandronate+BMP VS BMP	intraosseous	0.6 mg, once	8 weeks
Cheng TL	Traumatic	Zoledronic+BMP VS BMP	intraosseous	0.25 mg, once	8 weeks
Zou Y	Traumatic	Clodronate VS placebo Clodronate + Sim VS Sim	Intraosseous	2.2 mg, once	6 weeks
Aruwajoye OO	Traumatic	Ibandronate VS placebo BMP+Ibandronate VS BMP Zoledronic acid VS placebo	Intraosseous	0.6 mg, once	8 weeks
Little DG(1)	Traumatic	Zoledronic acid VS placebo	Subcutaneous	0.1 mg/kg/w × 3 w	6 weeks
Little DG(2)	Traumatic	Zoledronic acid VS placebo	Subcutaneous	0.15 mg/kg in total	15 weeks
Fan M	Traumatic	PLGA+zoledronate VS PLGA	Subcutaneous	0.1 mg/kg,once	5 weeks
Xu XL	Traumatic	Alendronate VS placebo	Implanted	30 μg, once	6 weeks
Hofstaetter JG	Traumatic	alendronate+Lova VS Lova	Subcutaneous	150 μg/kg, 3 × per/w × 2 w	24 weeks
Xie XW	Steroid	alendronate VS control	Oral	150 μg/(kg.d) × 4 w	12 weeks
Tan G	Steroid	alendronate VS control	Oral	150 μg/(kg.d) × 4 w	12 weeks
Xin DS(1)	Traumatic	alendronate VS control	Intragastric	1 mg/(kg.d) × 2 w	5 weeks
Xin DS(2)	Traumatic	alendronate VS control	Intragastric	1 mg/(kg.d) 2 w	5 weeks
Clinical studies					
Wang CJ	Alcohol, steroid	ESWT+alendronate VS ESWT	Oral	70 mg/w for 1 year	25 months
Chen CH	Alcohol, steroid	Alendronate +Ca+vitD VS Ca+vit D	Oral	70 mg/w for 2 year	24 months
Nishii T	Alcohol,steroid, Idiopathic	Alendronate VS placebo	Oral	5 mg/day for 1 year	12 months
Lai KA	Alcohol,steroid, Idiopathic	Alendronate VS placebo	Oral	70 mg/w for 25 week	24 months
Lee YK	Alcohol,steroid, Idiopathic	Zoledronate+Ca+vitD VS Placebo+Ca+vit D	Intravenous	5 mg/year for 2 year	24 months
Kang P	Alcohol,steroid, Idiopathic	Alendronate+MD+Ca+vitD VS PlaceboMD+Ca+vit D	Oral	10 mg/day or 70 mg/w for 24 weeks	63 months
Gao Y	steroid	alendronate+Ca VS placebo +Ca	Oral	70 mg/week for 6 month	6 months

BMP: Bone Morphogenetic Protein; PLGA: Poly (lactic-co-glycolic acid); sim: Simvastatin; Lova: Lovastatin; ESWT: extracorporeal shockwave therapy.

**Table 3:** The methodological quality of individual study.

**Authors**	**(1)**	**(2)**	**(3)**	**(4)**	**(5)**	**(6)**	**(7)**	**Total score**
Animal studies								
Kim HK(1)		*			*	*	*	4
Aya-ay J		*			*	*	*	4
Vandermeer JS		*			*	*		3
Kim HK (2)		*	*		*	*		4
Cheng TL		*	*		*	*	*	5
Zou Y		*	*		*	*		4
Aruwajoye OO		*	*		*	*		4
Little DG(1)		*	*		*	*		4
Little DG(2)		*			*	*	*	4
Fan M		*	*		*	*	*	5
Xu XL		*	*		*	*		4
Hofstaetter JG		*			*	*	*	4
Xie XW			*		*	*	*	4
Tan G			*		*	*	*	4
Xin DS(1)			*		*	*	*	4
Xin DS(2)			*		*	*	*	4

*One score means the studies fulfilling one of the criteria of (1) sample size calculation; (2) inclusion and exclusion criteria; (3) randomisation; (4) allocation concealment; (5) reporting of objects excluded from analysis; (6) blinded assessment; (7) reporting potential conflicts of interest and study funding.

**Table 4:** Modified Jadad Score for clinical trials.

Study	Randomization	Concealment of allocation	Double blinding	Total Withdrawals and dropouts	Total
Wang CJ	**		**	*	5
Chen CH	**	**	**	*	7
Nishii T	*	*	*	*	4
Lai KA	**		**	*	5
Lee YK	**	**	**	*	7
Kang P	**	*	**	*	6
Gao Y	**		**	*	5

Each asterisk represents one point. Modified Jadad score is used to evaluate the quality of articles and studies achieving a score of ≥4 points were considered to be of high quality.

### Primary analysis of animal studies

#### Evaluation of femoral head sphericity

**Figure 2:**
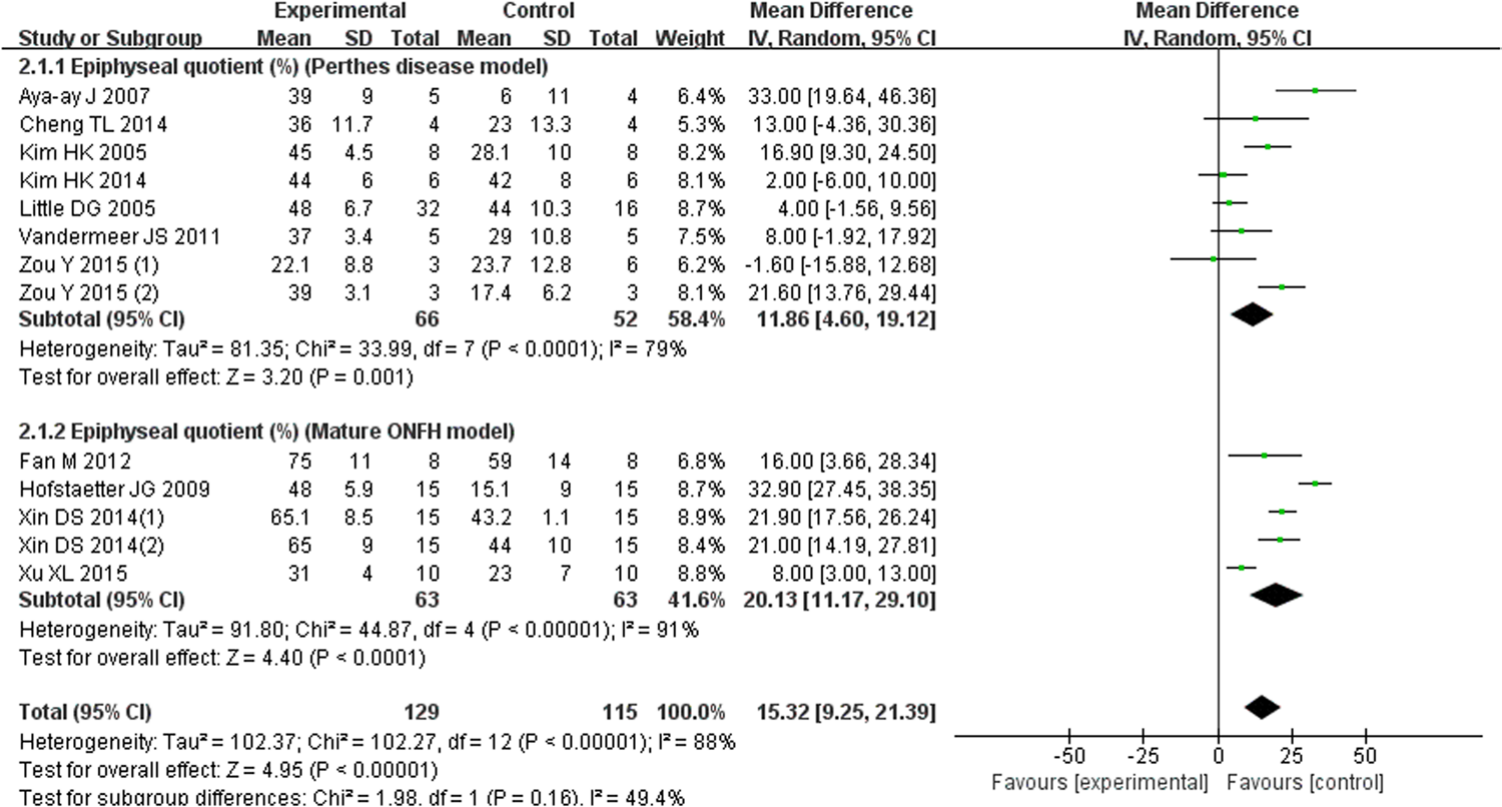
Forest plot showing Epiphyseal quotient.

Outcome evaluation and measurement methods are shown in Table 5. We found that the included animal studies recorded similar outcomes in the summary. Sphericity measurements of the femoral head were derived using a modified EQ, which indicated the height at the center of the femoral head over the width. Seven studies of 118 Perthes disease animal models recorded the EQ, which was significantly increased in the experimental group using bisphosphonates compared with the control group (MD = 11.86; 95% CI, 4.60–19.12; Fig. 2). Five studies of a mature ONFH model recorded the EQ, and the result indicated that the bisphosphonates treatment had better outcomes (MD = 20.13; 95% CI, 11.17–29.10; Fig. 2). The pooled results of the two animal models were also significantly better in the experimental group (MD = 15.32; 95% CI, 9.25–21.39; Fig. 2).

**Table 5:** Outcome evaluation and measurement method of animal studies.

Study	Outcome evaluation	Measurement method
Animal studies		
Kim HK(1)	EQ	X Radiographs
	Trabecular parameters (BV,TT,TN,TS)	Histomorphometry
Aya-ay J	EQ	X Radiographs
	Trabecular parameters (BV,TT,TN,TS)	Histomorphometry
Vandermeer JS	EQ	X Radiographs
	Trabecular parameters (BV,TT,TN,TS)	Histomorphometry
Kim HK (2)	EQ	X Radiographs
	Trabecular parameters (BV,TT,TN,TS)	Histomorphometry
Cheng TL	EQ	X Radiographs
Zou Y	EQ	X Radiographs
	Trabecular parameters (BV,TT)	Micro-Quantitative-CT
Aruwajoye OO	Trabecular parameters (BV)	Histomorphometry
Little DG(1)	Trabecular parameters (BV,TT,TN)	Histomorphometry
Little DG(2)	EQ	X Radiographs
	Trabecular parameters (BV,TT,TN)	Histomomhometrv
Fan M	EQ	X Radiographs
	Trabecular parameters (BV,TT,TS)	Micro-Quantitative-CT
Xu XL	EQ	X Radiographs
	Trabecular parameters (BV,TT,TS)	Histomorphometry
Hofstaetter JG	EQ	Micro- CT image
	Trabecular parameters (TT)	Micro-Quantitative-CT
Xie XW	Trabecular parameters (TT,TN,TS)	Micro-Quantitative-CT
Tan G	Trabecular parameters (TT,TN,TS)	Micro-Quantitative-CT
Xin DS(1)	EQ	X Radiographs
	Trabecular parameters (BV,TT,TN,TS)	Micro-Quantitative-CT
Xin DS(2)	EQ	X Radiographs
	Trabecular parameters (BV,TT,TN,TS)	Micro-Quantitative-CT

EQ: Epiphyseal quotients; BV: bone volume; TT: Trabecular thickness; TN: trabecular number; TS: Trabecular separation.

#### Parametric analysis of bone trabeculae

**Figure 3:**
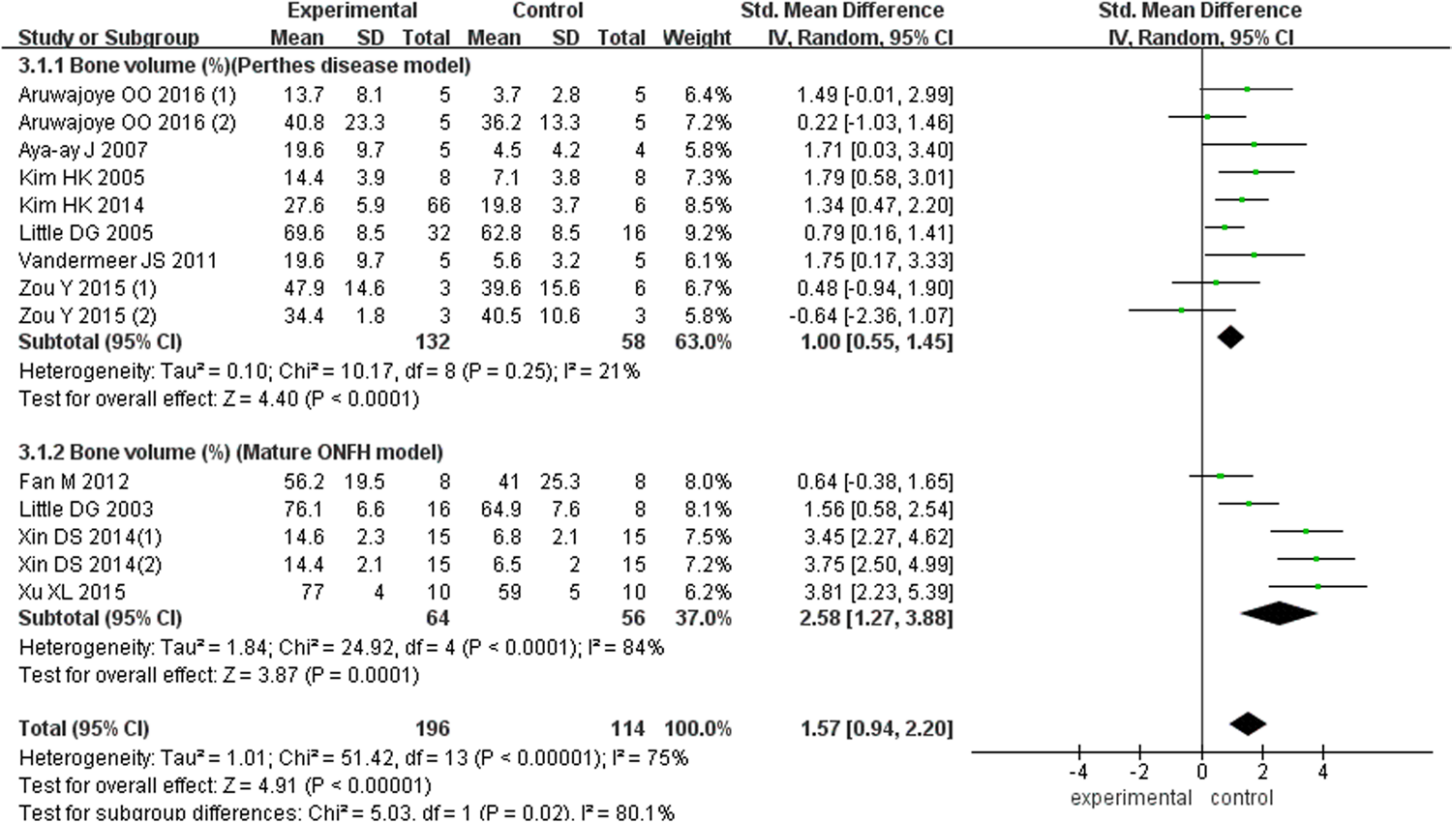
Forest plot showing bone volume.

Trabecular bone volume (BV/TV) was presented in seven articles involving 190 animals with Perthes disease, and this meta-analysis demonstrated that animals in the bisphosphonate group had greater BV improvement (MD = 1.00; 95% CI, 0.55–1.45). Five studies of 120 mature ONFH models showed better BV/TV performance in the experimental group (SMD = 2.58; 95% CI, 1.27–3.88). The pooled outcomes were also higher in the bisphosphonate group (SMD = 1.57; 95% CI, 0.94–2.20) (Fig. 3).

**Figure 4:**
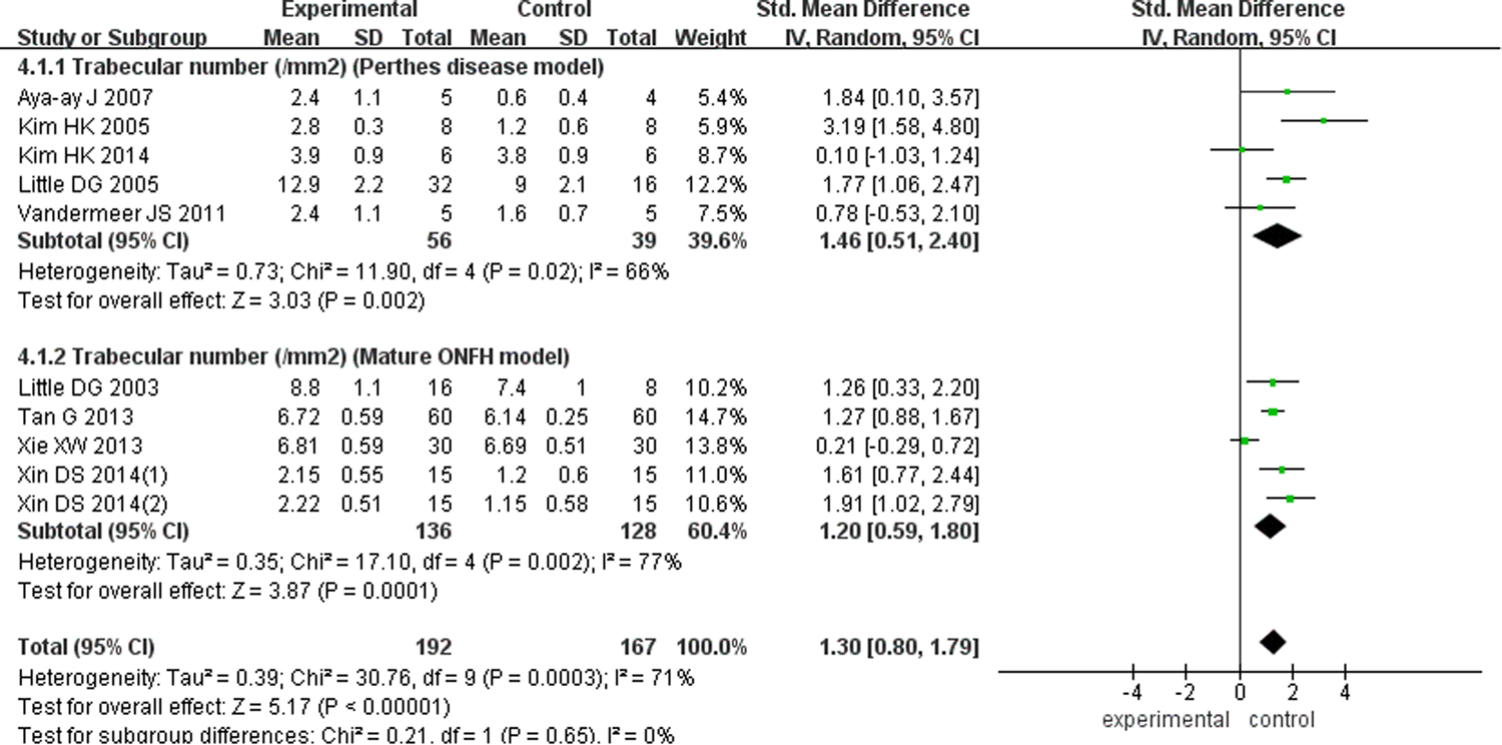
Forest plot showing trabecluar number.

**Figure 5:**
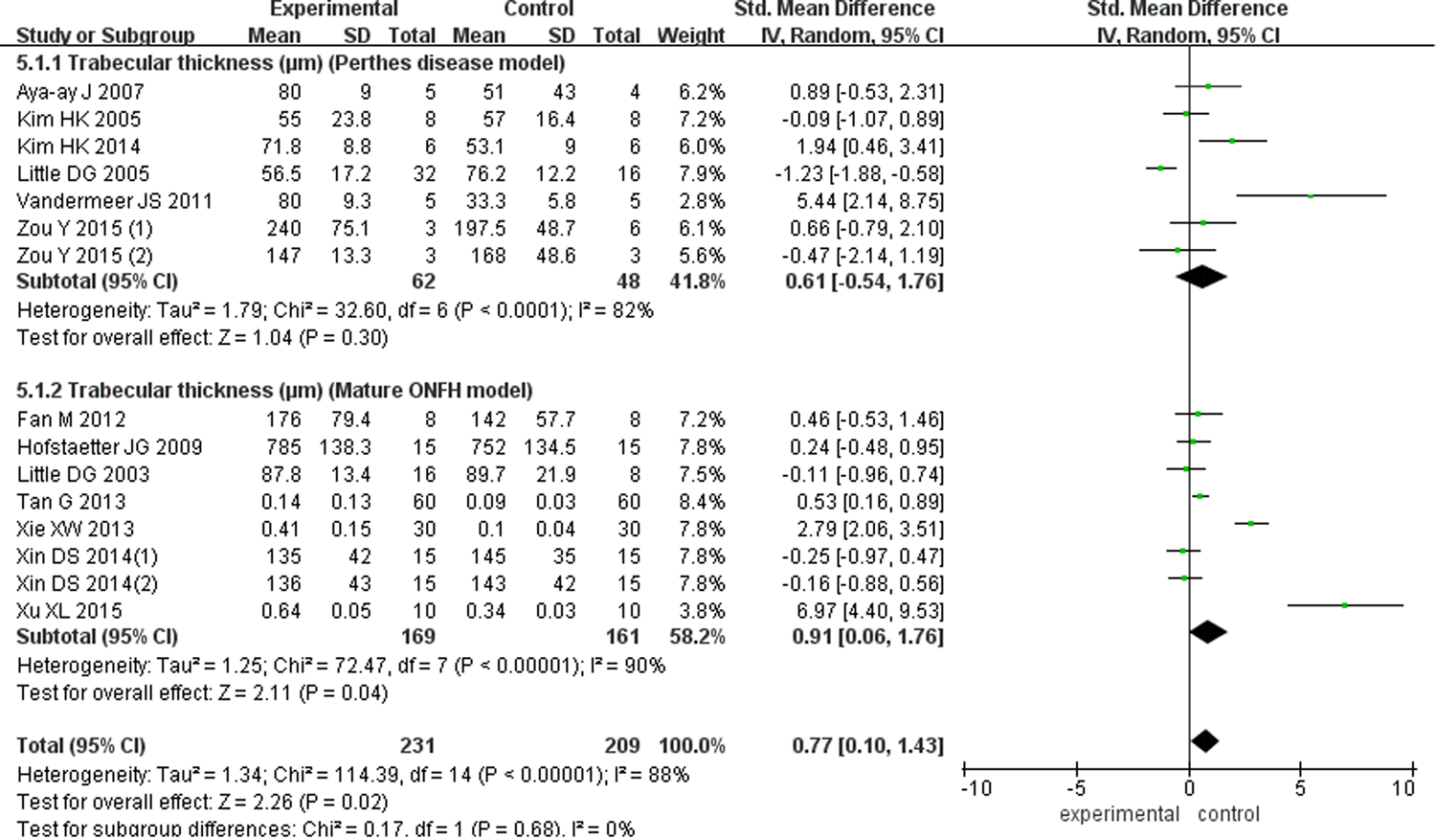
Forest plot showing trabecular thickness.

**Figure 6:**
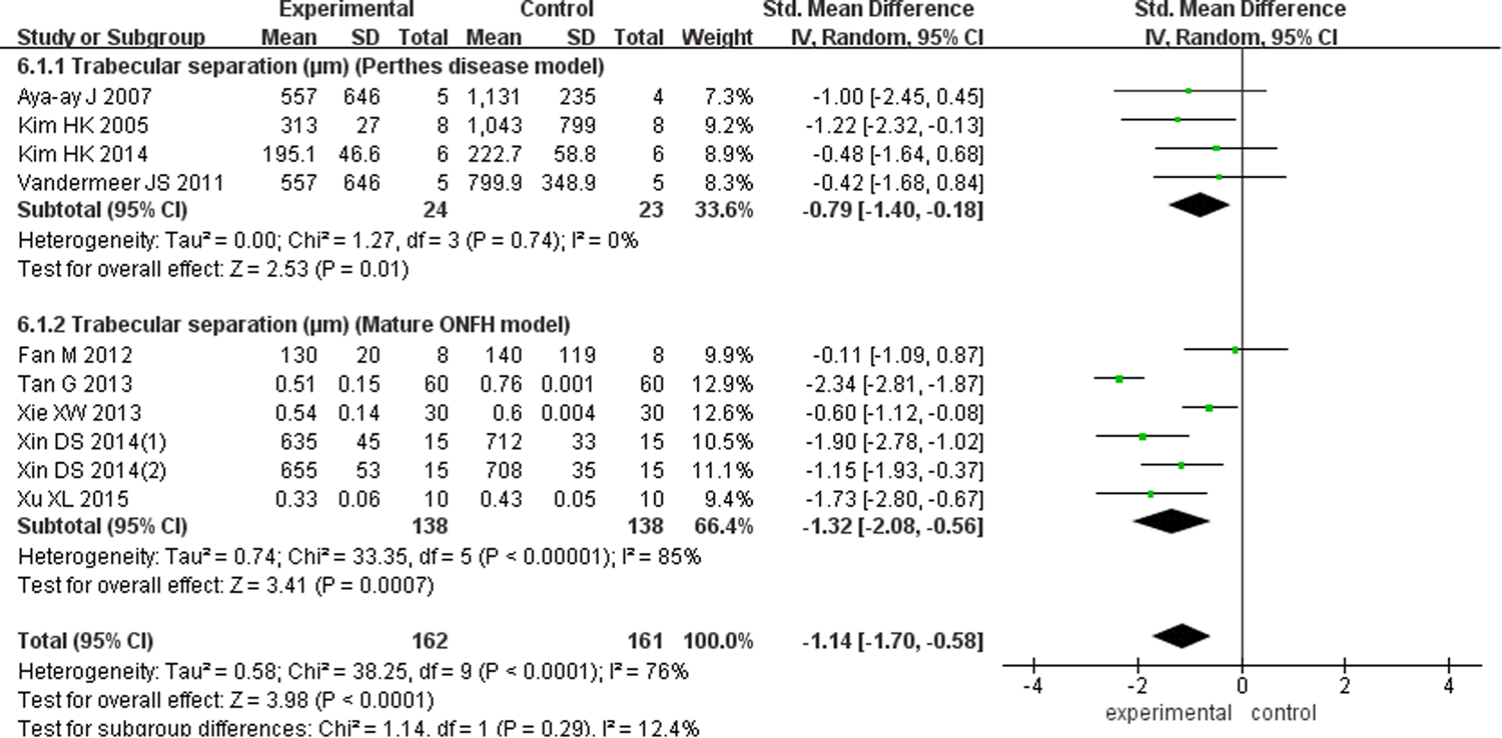
Forest plot showing trabecular separation.

There were five studies with 95 Perthes disease models (SMD = 1.46; 95% CI, 0.51–2.40) and five studies of 264 mature ONFH models (SMD = 1.20; 95% CI, 0.59–1.80) showed the TN in this meta-analysis, and according to the pooled outcomes, the experimental group was superior to the control group (SMD = 1.30; 95% CI, 0.80–1.79; Fig. 4). Six studies of 110 Perthes disease models (SMD = 0.61; 95% CI, −0.54–1.76) and another eight studies of 330 mature ONFH models (SMD = 0.91; 95% CI, 0.06–1.76) analyzed the trabecular thickness and reported significantly better results in the bisphosphonate group (SMD = 0.77; 95% CI, 0.10–1.43; Fig. 5). At the same time, four articles examining 47 Perthes disease models (SMD = −0.79; 95% CI, −1.40 to −0.18) and six studies of 276 mature ONFH models (SMD = −1.32; 95% CI, −2.08 to −0.56) showed that the bisphosphonates improved the trabecular separation (SMD = −1.14; 95% CI, −1.70 to −0.58; Fig. 6).

#### Outcomes of clinical trials

**Figure 7:**
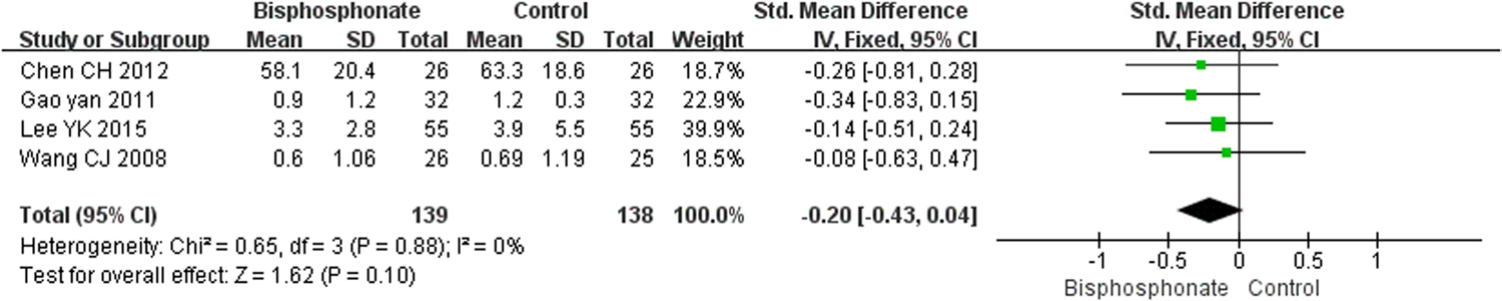
Forest plot showing pain score.

**Figure 8:**
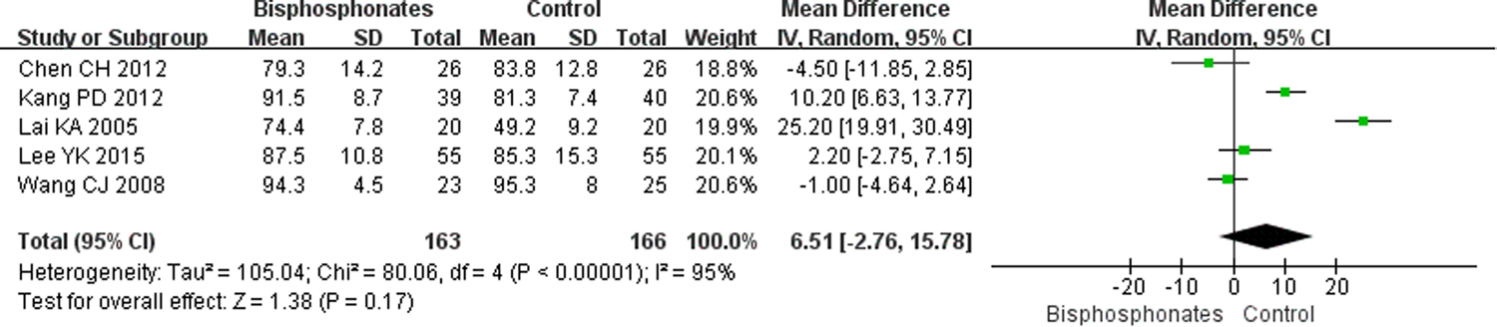
Forest plot showing Harris score.

**Figure 9:**
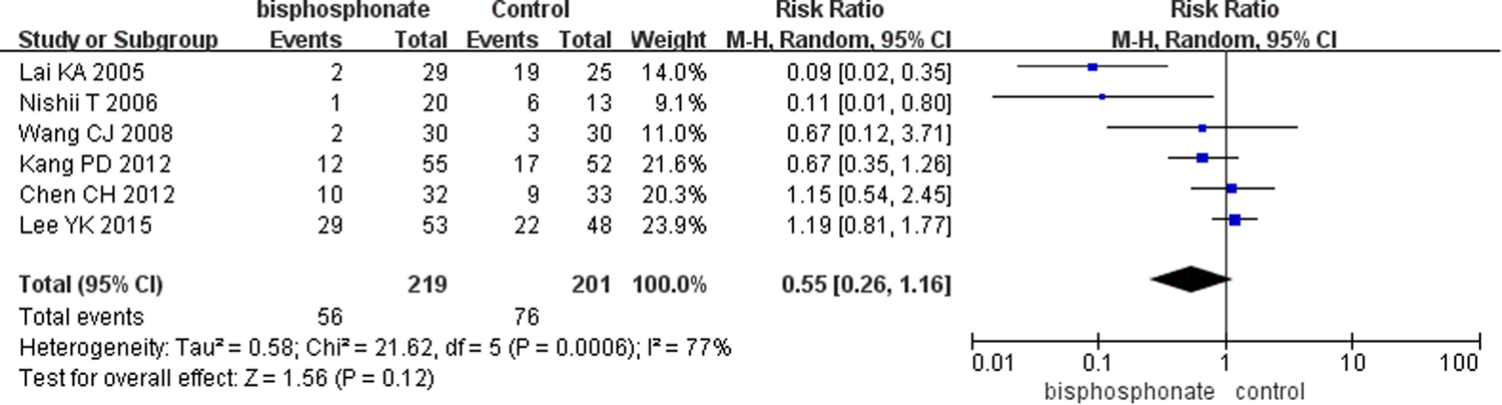
Forest plot showing collapse of the femoral head.

**Figure 10:**
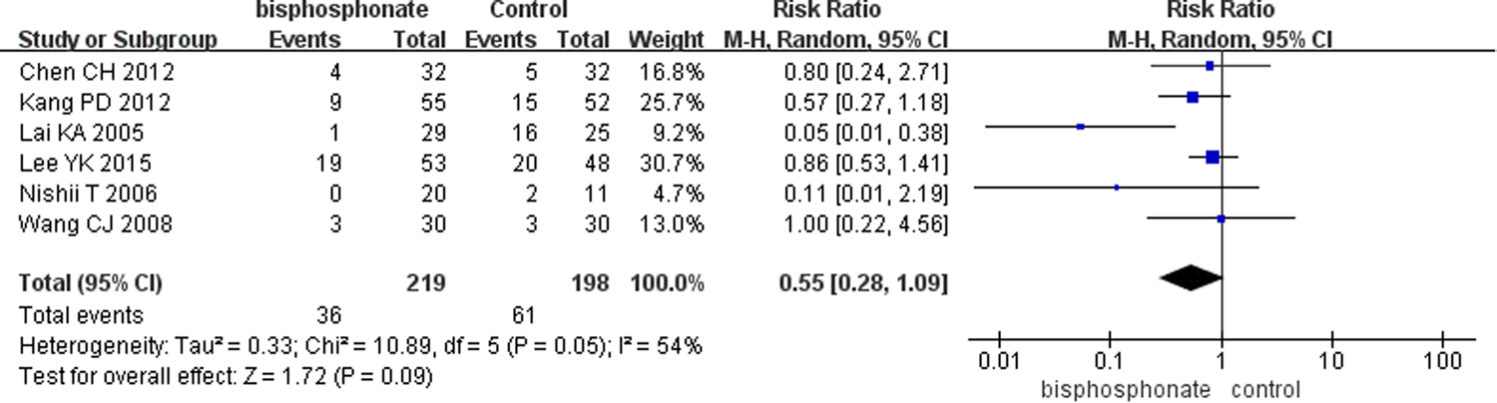
Forest plot showing patients undergoing total hip arthroplasty.

The pooled results of pain score with four studies of 277 patients and the hip Harris scores from five studies of 329 patients showed that bisphosphonate use achieved better pain scores (SMD = −0.20; 95% CI, −0.43–0.04; Fig. 7) and higher Harris scores (MD = 6.51; 95% CI, −2.76–5.78; Fig. 8); however, the differences were not statistically significant (p = 0.10 and p = 0.17, respectively). At the same time, the overall estimated proportion of patients who experienced progression to collapse in six studies involving 420 cases seemed to be reduced by bisphosphonate therapy (RR = 0.55; 95% CI, 0.26–1.16; Fig. 9), but it no significant difference was noted (p = 0.12). Likewise, the THA incidence tended to improve in patients treated with bisphosphonates (RR = 0.55; 95% CI, 0.28–1.09; Fig. 10), but the difference was not statistically significant (p = 0.09).

## Discussion

This meta-analysis aimed to determine whether bisphosphonates exerted effects on preventing femoral head collapse after osteonecrosis in animal models or clinical trials. Our results showed that bisphosphonate use significantly improved EQ indicative of femoral head sphericity as well as better BV, TN, trabecular separation, and trabecular thickness in the animal model. Unexpectedly, this finding is not supported by clinical studies, which showed no statistically significant differences in pain improvement, complications, or the need for THR. The animal studies and clinical trials seemed to present discordant outcomes.

There is a balance between osteoblast and osteoclast activity in the repair of ONFH 8^41,42^. Osteoblasts are responsible for new bone formation, while osteoclasts are the bone resorptive cells. Although removal of the dead bone is also beneficial to the body, an excessively increased resorption rate leads to femoral head deformity and collapse. Some researchers have found that the osteoclasts become more active and develop a longer lifespan in the presence of osteonecrosis^43,44^. This may be the cause of the imbalance between osteogenesis and bone resorption. Bisphosphonates suppress the HMG-CoA reductase pathway and then inhibit osteoclast-mediated bone resorption, both of which accelerate osteoclast death^32^.

Although many animal studies^16,17,27^ and clinical trials^14,15^ have proven the efficiency of bisphosphonates in the treatment of ONFH, other researchers maintain different opinions. In clinical studies, Lee YK, *et al*. used zoledronate to treat patients with Steinberg stage I or II ONFH with a medium to large necrotic area, but their outcomes show that zoledronate does not prevent collapse of the femoral head or reduce the need for total hip arthroplasty^39^. Chen CH, *et al*. conducted a multicenter, prospective, randomized, double-blind, placebo-controlled study using alendronate to prevent femoral head collapse but concluded that alendronate had no obvious effects on decreasing the need for THA and cannot reduce disease progression or improve quality of life^21^. Moreover, the animal studies of Aruwajoye OO, *et al*.^19^ and Zou Y, *et al*.^18^ showed that the use of ibandronate alone did not obviously improve osteonecrosis, while the combination of ibandronate and other drugs such as BMP-2 or simvastatin could exert better protective effects. Likewise, Fan, *et al*.^30^ demonstrated that zoledronate could inhibit the formation of new vasculature, which may not benefit the repair process of ONFH. Accordingly, the current data was in controversy on the effectiveness of bisphosphonates.

Based on the outcomes of this meta-analysis, we found the EQ that stands for the height at center of the femoral head over the width, which used to evaluate the femoral head sphericity, was improved in the bisphosphonate group. This means that the using of bisphosphonates indeed exert effects on protecting the femoral head morphology. At the same time, the BV, TN, trabecular thickness, and trabecular separation factors used to assess bone mass of the femoral head in the animal model were all significantly improved by bisphosphonate use, a finding that was very encouraging. These results indicated that controlling the pathological activity of the osteoclasts helped repair the ONFH^30^. At the same time, some other studies^17,45^ reported that inhibiting osteoclast activity would help increase osteoblast action and, in turn, lead to a positive balance of bone formation. Resorption of dead compact bone during osteonecrosis repair may decrease the structural properties and mechanical support of the femoral head and may be partially responsible for the collapse in the late stages of osteonecrosis^17,45^. Thus, intervening in this process with bisphosphonates would make some difference just as our results showed. However, we also realized that those encouraging outcomes were drawn from the heterogeneous methods/models. Animal models of ONFH or Perthes disease are still too heterogeneous in terms of animal type, age, sex, interventionist strategy, and duration. The methods using bisphosphonates to treat ONFH also varied, including different bisphosphonate types, administration approaches, dosage, and durations, which may have influenced the outcomes. In summary, we observed improvement of the structural changes in terms of bone architecture and BV, but whether it could treat the ONFH in the animal model remains difficult to judge based on our outcomes alone.

Translating the animal results into clinical outcomes is always not easy, as discordant findings are usually present^46,47^. Although heterogeneous animal models suggest the improvement of some bone morphology in this meta-analysis, this is not supported by pooled clinical studies, which showed no statistical differences in pain improvement, complications, or the need for THR. In fact, the clinical studies also showed high heterogeneity, including different bisphosphonate drugs, administration approaches, and combination with many other procedures, all of which may contribute to different outcomes present in those studies and also influence the pooled outcomes in this meta-analysis. Selection bias in terms of patient case mix, sample bias, publication bias, or unintended bias due to the interpretation of post-randomization events could also have changed the outcomes^46^.

However, the obviously discordant findings in animal and human studies were still associated with some reasons. Animals and humans have different sensitivities to bisphosphonates. Animals were usually given adequate treatment doses throughout life, whereas humans were usually given low doses. It is easier to analyze the femoral head in animal models, both with radiographic films and specimen experiments. We only needed to analyze the desired outcomes while ignoring many other adverse events in animals. When we evaluated the patients, it was not until the femoral head had collapsed that we could obtain samples after the THA surgery, which means that the analytical methods were completely different and an effective evaluation method is lacking for human subjects. Besides, medication use in humans considers more consideration of safety, complications, and the overall body condition, all factors of which made the process differ from that of the animal experiment. More importantly, a poor methodology, publication bias, and inadequate animal models that simply do not reflect human disease are to blame for the discordant findings. Last but not least, it is difficult to know whether animal studies face similar problems regarding trial design. Clinical trials are usually powered to predict the number of patients likely to generate a statistical difference for the primary population. Additionally, to eliminate bias, patients are randomized and treatments are administered blindly by the researcher. In contrast, animal experiments are unlikely to be powered (usually concentrating on a static number per group according to laboratory preference) and rarely report crucial experimental design factors such as randomization and blinding^46^. To this end, the lack of bias elimination in preclinical studies is thought to contribute to a five-fold likelihood of showing a beneficial therapeutic effect^48^. Thus, using standardized animal models and standardized experiment methods may eliminate some of the differences between animal and human studies, enabling a greater degree of translation.

## Conclusion

Bisphosphonates could improve bone architecture and BV in animal studies, but those results did not translate into either symptomatology or end-stage complications and management in the human studies. This might suggest that poor methodology, publication bias, and inadequate animal models that simply do not reflect human disease are to blame for the discordant findings. Thus, systematic reviews of animal studies are needed to ensure that the findings will be relevant to the design of clinical trials, while the use of standardized animal models and standardized experiment methods may eliminate some of the differences between animal and human studies to enable a greater degree of translation.

## References

[1] Hungerford DS, Jones LC (2004). Asymptomatic osteonecrosis: should it be treated?. Clin. Orthop. Relat. Res..

[2] Mankin HJ (1992). Nontraumatic necrosis of bone (osteonecrosis). N. Engl. J. Med..

[3] Kealey WD (2000). Deprivation, urbanisation and Perthes’ disease in Northern Ireland. J. Bone. Joint. Surg. Br..

[4] Kim HK (2012). Pathophysiology and new strategies for the treatment of legg-calve-perthes disease. J. Bone. Joint. Surg..

[5] Stulberg SD, Cooperman DR, Wallensten R (1981). The natural history of Legg-Calve-Perthes disease. J. Bone. Joint. Surg. Am..

[6] Kim SY (2004). Multiple drilling compared with core decompression for the treatment of osteonecrosis of the femoral head. J. Bone. Joint. Surg. Br..

[7] Chang CC, Greenspan A, Gershwin ME (1993). Osteonecrosis: current perspectives on pathogenesis and treatment. Semin. Arthritis. Rheum..

[8] Catterall A (1982). Perthes’ disease: is the epiphysial infarction complete?. J. Bone. Joint. Surg. Br..

[9] Allen MR, Burr DB (2011). Bisphosphonate effects on bone turnover, microdamage, and mechanical properties: what we think we know and what we know that we don’t know. Bone..

[10] Pazianas M (2014). Bisphosphonates and bone quality. BoneKey. Rep..

[11] Silverman S, Christiansen C (2012). Individualizing osteoporosis therapy. Osteoporos. Int..

[12] Reid IR (1996). Biochemical and radiologic improvement in Paget’s disease of bone treated with alendronate: a randomized, placebo-controlled trial. Am. J. Med.

[13] Lane JM (2001). Bisphosphonate therapy in fibrous dysplasia. Clin. Orthop. Relat. Res..

[14] Kang P (2012). Are the results of multiple drilling and alendronate for osteonecrosis of the femoral head better than those of multiple drilling? A pilot study. Joint. Bone. Spine..

[15] Lai KA (2005). The use of alendronate to prevent early collapse of the femoral head in patients with nontraumatic osteonecrosis. A randomized clinical study. J. Bone. Joint. Surg. Am..

[16] Little DG (2003). Zoledronic acid treatment results in retention of femoral head structure after traumatic osteonecrosis in young Wistar rats. J. Bone. Miner. Res..

[17] Hofstaetter JG (2009). The effects of alendronate in the treatment of experimental osteonecrosis of the hip in adult rabbits. Osteoarthritis. Cartilage..

[18] Zou Y (2015). Synergistic local drug delivery in a piglet model of ischemic osteonecrosis: a preliminary study. J. Pediatr. Orthop. B..

[19] Aruwajoye OO, Aswath PB, Kim HK (2017). Material properties of bone in the femoral head treated with ibandronate and BMP-2 following ischemic osteonecrosis. J. Orthop. Res..

[20] Wang CJ (2008). Treatment of osteonecrosis of the hip: comparison of extracorporeal shockwave with shockwave and alendronate. Arch. Orthop. Trauma. Surg..

[21] Chen CH (2012). Alendronate in the prevention of collapse of the femoral head in nontraumatic osteonecrosis: a two-year multicenter, prospective, randomized, double-blind, placebo-controlled study. Arthritis. Rheum..

[22] Yuan HF, Guo CA, Yan ZQ (2016). The use of bisphosphonate in the treatment of osteonecrosis of the femoral head: a meta-analysis of randomized control trials. Osteoporos. Int..

[23] Moher D (2009). Preferred reporting items for systematic reviews and meta-analyses: the PRISMA statement. Plos. Med..

[24] Fisher M (2009). Update of the stroke therapy academic industry roundtable preclinical recommendations. Stroke..

[25] Oremus M (2001). Interrater reliability of the modified Jadad quality scale for systematic reviews of Alzheimer’s disease drug trials. Dement. Geriatr. Cogn. Disord..

[26] Kim HK (2005). Ibandronate for prevention of femoral head deformity after ischemic necrosis of the capital femoral epiphysis in immature pigs. J. Bone. Joint. Surg. Am..

[27] Aya-ay J (2007). Retention, distribution, and effects of intraosseously administered ibandronate in the infarcted femoral head. J. Bone. Miner. Res..

[28] Vandermeer JS (2011). Local administration of ibandronate and bone morphogenetic protein-2 after ischemic osteonecrosis of the immature femoral head: a combined therapy that stimulates bone formation and decreases femoral head deformity. J. Bone. Joint. Surg. Am..

[29] Kim HK (2014). Local administration of bone morphogenetic protein-2 and bisphosphonate during non-weight-bearing treatment of ischemic osteonecrosis of the femoral head: an experimental investigation in immature pigs. J. Bone. Joint. Surg. Am..

[30] Cheng TL (2014). Local delivery of recombinant human bone morphogenetic proteins and bisphosphonate via sucrose acetate isobutyrate can prevent femoral head collapse in Legg-Calve-Perthes disease: a pilot study in pigs. Int. Orthop..

[31] Little DG (2005). Zoledronic acid improves femoral head sphericity in a rat model of perthes disease. J. Orthop. Res..

[32] Fan M (2012). Effect and mechanism of zoledronate on prevention of collapse in osteonecrosis of the femoral head. Zhongguo. Yi. Xue. Ke. Xue. Yuan. Xue. Bao..

[33] Xu XL (2015). Prevention of femoral head collapse due to osteonecrosis in rats by local delivery of zoledronate via poly lactic-co-glycolic acid. Orthopedic. Journal. Of. China..

[34] Xie XW (2013). Effects of alendronate and lovastatin in preventing early glucocorticoids-induced osteonecrosis of femoral head in rats by micro-CT. Orthopedic. Journal. of.China..

[35] Tan G (2013). Micro-CT evaluation of alendronate in preventing glucocorticoids-induced osteonecrosis of femoral heads of rats. Chin. J. Joint. Surg. (Electronic Edition)..

[36] Xin DS (2014). Constructing a rat model of traumatic osteonecrosis of the femoral head with articular surface collapse and prevention mechanism of alendronate. Chinese. Journal. of.Tissue. Engineering. Research..

[37] Xin DS (2014). Effects of strontium ranelate on the prevention of collapse in traumatic osteonecrosis of the femoral head in rats using micro-computed tomography. Orthopedic. Journal. of. China..

[38] Nishii T (2006). Does alendronate prevent collapse in osteonecrosis of the femoral head?. Clin. Orthop. Relat. Res..

[39] Lee YK (2015). Does Zoledronate Prevent Femoral Head Collapse from Osteonecrosis? A Prospective, Randomized, Open-Label, Multicenter Study. J. Bone. Joint. Surg. Am..

[40] Gao Y (2016). Clinical Observation of Early Steroid Secondary Osteonecrosis of Alendronate Therapy. Guide. Of. China. Medicine..

[41] Terayama H (2011). Prevention of osteoneerosis by intravenous administration of human peripheral blood-derived CD34-positive cells in a rat osteonecrosis model. J. Tissue. Eng. Regen. Med..

[42] Li W (2009). Distribution of TRAP-post-tire cells and expression of HIF-l alpha, VEGF, and FGF-2 in the reparative reaction in patients with osteonecrosis of the femoral head. J. Orthop. Res..

[43] Weinstein RS (2010). Endogenous glucocorticoids decrease skeletal angiogenesis,vascularity,hydration,and strength in aged mice. Aging. Cell..

[44] Nagashima M (2005). Bisphosphonate (YM529) delays the repair of cortical bone defect after drill-hole injury by reducing terminal differentiation of osteoblasts in the mouse femur. Bone..

[45] Hofstaetter JG (2006). Changes in bone microarchitecture and bone mineral density following experimental osteonecrosis of the hip in rabbits. Cells. Tissues. Organs..

[46] Dyson A, Singer M (2009). Animal models of sepsis: why does preclinical efficacy fail to translate to the clinical setting?. Crit. Care. Med..

[47] Krug N, Rabe KF (2008). Animal models for human asthma: the perspective of a clinician. Curr. Drug. Targets..

[48] Perel P, Roberts I, Sena E (2007). Comparison of treatment effects between animal experiments and clinical trials: Systematic review. B.M.J..

